# Electrodeposition, microstructure and characterization of high-strength, low-roughness copper foils with polyethylene glycol additives†

**DOI:** 10.1039/d4ra06688j

**Published:** 2024-12-03

**Authors:** Jian Huang, Ning Song, Mingwei Chen, Yunzhi Tang, Xiaowei Fan

**Affiliations:** a Jiangxi Province Key Laboratory of Functional Crystalline Materials Chemistry, School of Chemistry and Chemical Engineering, Jiangxi University of Science and Technology Ganzhou 341000 China tangyunzhi75@163.com

## Abstract

Electroplating additives play a key role in enhancing the physical and chemical performances of electrodeposited copper foils. Currently, a lot of research on polyethylene glycol (PEG) additives has been reported on the metallisation interconnections for printed circuit boards (PCBs); however, research on its applications for electrodeposited copper foils is rarely documented. Herein, high-quality copper foils with high tensile strength (433.2 MPa) and low surface roughness (Rz = 1.7 μm) were electrodeposited on titanium substrates with 4 mg L^−1^ PEG. The characterization results showed that the copper foil possesses a high (220) crystal plane orientation and low twin layer thickness. Further analysis suggested that twin boundaries strengthened by stacking faults were the main reason for the high tensile strength of copper foils. Electrochemical behavioral studies indicated that PEG increased the cathodic polarization and improved the nucleation rate, thereby refining the copper foil grain size. The high (220) crystal plane orientation and high density of twin grain boundaries caused by the stacking faults strengthened the copper foil more than the fine-grain strengthening. This work offers theoretical guidance for modulating the properties of copper foil using PEG.

## Abstract 2

High-quality copper foils with high tensile strength (433.2 MPa) and low surface roughness (1.7 μm) were electrodeposited with PEG, exhibiting high (220) crystal plane orientation, high density of twin grain boundaries and stacking faults.

## Graphical abstract

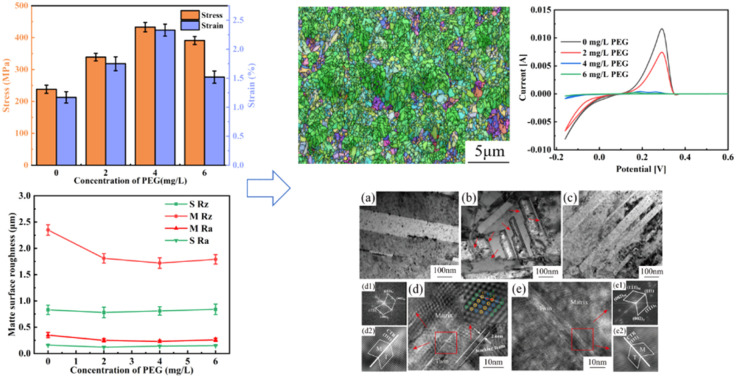

## 1. Introduction

Electrolytic copper foils have a wide range of applications in the manufacturing of printed circuit boards (PCBs) due to their excellent electrical and thermal conductivities. In recent years, printed circuit boards have been developed to meet high density, high speed and high reliability requirements.^1–3^ High-strength copper foils can withstand large tensions, thus ensuring the stability and reliability of PCBs.^4^ Therefore, copper foils need to have excellent mechanical properties to ensure that they do not break or fail under stress. Also, a rough copper foil surface could increase the loss of high-frequency signal transmission in PCBs.^5–8^ Therefore, it is important to prepare copper foils with excellent mechanical properties and low surface roughness to meet the demands for high-frequency, high-speed PCBs.

Numerous studies have shown that additives can play a key role in the performance regulation of electrodeposited copper foils. Trace additives in the plating solution mainly affect the electrochemical reaction process at the electrode interface through cathodic adsorption,^9–11^ metal ion complexation^12–15^ and ion diffusion,^16,17^ thus regulating the properties of copper foils.

PEG is a typical copper plating inhibitor. The inhibitory effect of PEG is realised by the adsorption of complexes on the cathode surface, hindering the reduction of Cu^2+^;^18^ Cl^−^ enhances the inhibitory effect on Cu^2+^ reduction.^18–20^ Surface-enhanced Raman spectroscopy confirmed the molecular structure of the complexes as PEG–Cu^+^–Cl^−^ complexes.^21^ Wang *et al.*^22^ investigated the effect of temperature on Cl^−^ adsorption on the copper surface and on the PEG–Cu^+^–Cl^−^ structure. The results showed that the inhibition effect of PEG was negatively correlated with the working temperature. Lin *et al.*^23^ found that the adsorption of PEG molecules not only inhibited the deposition rate of copper but also inhibited hydrogen precipitation. In addition, Ko *et al.*^18^ found that the inhibition ability of the PEG–Cu^+^–Cl^−^ complexes was affected by the molecular weight of the PEG and that this effect was positively correlated with the molecular weight of PEG within a certain range. However, the current study mainly focused on the effect of PEG on the copper filling in multilayer PCB microperforation plating. There is a dearth of studies on the role of PEG in the preparation of electrodeposited copper foils, especially its systematic effects on the resulting copper foils' microstructure and mechanical properties under high current density, high acid concentration, and high copper concentration.

This study investigated the organisation and structure of copper foils and the behavior of PEG in electrochemical reactions to uncover the effects and mechanism of action of PEG on the organization and mechanics of the foils. On this basis, copper foils with good mechanical properties and smooth surfaces were prepared. This study offers theoretical guidance for regulating the properties of copper foil using PEG and for preparing high-performance copper foils.

## 2. Experimental section

A self-designed plate electrodeposition device was fabricated and used in all the experiments. This device comprised an electrolytic cell, an electroplating bath stirring system, and a temperature monitor and controller system. Pure titanium plates and titanium plates coated with iridium were used as the cathode and anode, respectively, and the distance between the electrodes was fixed at 5 cm. Air was blown into the plating solution at a flow rate of 20 L min^−1^ during the electrodeposition process to promote sufficient diffusion of the copper ions and additives. The temperature control system maintained the plating bath temperature at 50 °C ± 1 °C. Prior to each experiment, the titanium cathode plates were mechanically polished to a roughness of Rz <1 μm and then washed sequentially in ultrasonic baths of acetone, ethanol, and water for 5 min, followed by deionised water.

All the reagents used in the experiment were of analytical grade. Table 1 lists the main components of the electroplating bath. Deionized water was used as the experimental water. Fresh solutions were prepared before each experiment to prevent degradation over time. The electrodeposition process was conducted under a constant current density of 15 A dm^−2^. The electrodeposition time was adjusted to achieve a nominal copper foil thickness of 12 μm. The thickness of the prepared copper foil samples was measured using a handheld film thickness gauge (HITACHI-CMI165). The results indicated that all the samples had a thickness of 12 μm, with a thickness deviation of only 0.1 μm. After deposition, the copper foils were carefully removed from the cathode, rinsed with deionised water, and then air-dried.

**Table 1:** Electroplating bath composition

Component	A	B	C	D
CuSO_4_ g L^−1^	80	80	80	80
H_2_SO_4_ mL L^−1^	66	66	66	66
HCl μL L^−1^	47	47	47	47
PEG mg L^−1^	—	2	4	6

The copper foil surface facing the titanium plate is referred to as the shiny surface, while the surface exposed to the electroplating bath is called the matte surface. The morphology of the matte surface of the copper foil was characterised by scanning electron microscopy (SEM; MLA650F, FEI) operated at a voltage of 20 kV. The 3D information was obtained using a 3D optical profiler (Bruker Countor GT K 3D). Transmission electron microscopy (TEM; Talos F200X, FEI) was used to observe the internal microstructure and morphology of the copper foils. The microstructure of the copper foil was further analysed by high-resolution transmission electron microscopy (HRTEM) and selected area electron diffraction (SAED). Additionally, an electron backscatter diffraction (EBSD, Nordlys max3) system affixed to a SEM instrument (EDAX, TSL) was employed for EBSD characterisation of the copper foil. The cross-section of the copper foil was prepared using an argon ion cutting instrument (MODEL-1061). Initially, cutting was performed at 8 kV for 1 h, followed by a fine-tuning process at 3 kV for 0.5 h. Liquid nitrogen was used throughout the cutting process to cool the sample, thereby preventing thermal effects from occurring. Grain orientation maps, grain size statistics, and twin boundary maps of the copper foils were calculated using AZtecCrystal 2.1 software. X-Ray diffraction (XRD, TD3500, Tongda) was utilised to determine the crystal structure of the copper foil. All the samples underwent performance and structural testing within a short period, thereby avoiding the effects of self-annealing in the copper foil (ESI, Fig. S1†). The texture coefficient (*M*_*hkl*_), which reflects the preferred orientation of the matte surface of the copper foil, is defined as:^24^1
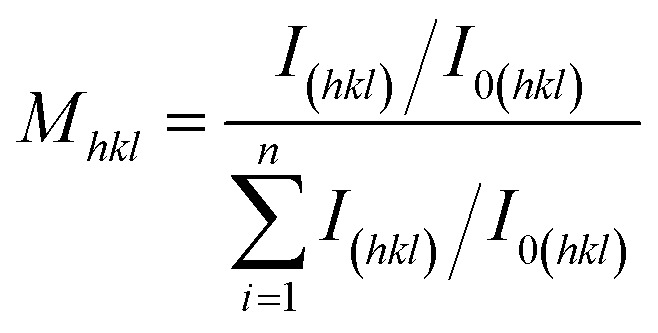
where *I*_(*hkl*)_ and *I*_0(*hkl*)_ are the relative peak intensity from the (*hkl*) reflection of an electrodeposited copper foil and that of the randomly oriented standard copper sample, and *n* is the number of calculated peaks.

The tensile strength and elongation of the copper foil were measured using a universal mechanics experimental machine (EM6.203, TSMT) at room temperature. The rectangular specimen's dimensions were 150 mm × 12.7 mm, the tensile testing machine scale was set at 50 mm, the strain rate was kept constant at 2 mm min^−1^, and the tensile test was carried out according to the standard set in GB/T 29847-2013. The surface roughness of the copper foil was measured using a roughness meter (PS-10, MarSurf). Five different positions were tested on each sample and the average value was taken as the final measurement. Electrochemical measurements were performed using an electrochemical workstation (660E, CHI) with a platinum sheet (1.5 cm^2^) for the counter electrode, a saturated mercurous sulfate electrode (SSE) as the reference electrode, and a titanium disk electrode (*Φ* 2 mm) as the working electrode. Prior to each measurement, the working electrode was polished with alumina paste, cleaned with deionised water, and then dried.

## 3. Results and discussion

### 3.1 Properties of the copper foil

**Fig. 1:**
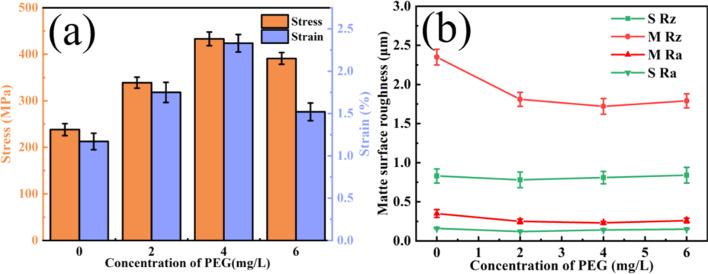
SEM images of the surface of copper foils prepared with different concentrations of PEG: (a) 0 mg L^−1^, (b) 2 mg L^−1^, (c) 4 mg L^−1^, (d) 6 mg L^−1^.

Fig. 1a shows the histograms of the tensile strengths and elongations of the copper foils prepared with various PEG concentrations. As the figure shows, the tensile strength of the copper foil increased and then decreased as the PEG concentration increased. The peak tensile strength of the copper foil prepared with a PEG concentration of 4 mg L^−1^ was 433.5 MPa, and the elongation was 2.2%. The tensile strength and elongation were increased by 82% and 46%, respectively, compared with the copper foil without PEG. When the PEG concentration was further increased to 6 mg L^−1^, the tensile strength and elongation of the copper foils decreased significantly, to 391 MPa and 1.5%, respectively.

Fig. 1b shows the variation curves of the shiny (S Rz and S Ra) and matte (M Rz and M Ra) surface roughness copper foils prepared with different concentrations of PEG added to the plating solution. The roughness parameters Ra and Rz are the key indicators used to characterise the surface roughness properties of copper foils. Ra represents the average of the absolute values of the distances from each point on the contour to the midline, while Rz indicates the distance between the highest and lowest points of the contour. As the figure shows, the Rz value remained relatively stable as the PEG concentration increased. Since the roughness of the shiny surface is only related to the roughness of the titanium plate, it was unaffected by the additives or electrochemical conditions.^25^ Therefore, the roughness of the copper foil mainly refers to the roughness of the matte surface. The variations in the matte surface's roughness are discussed in the subsequent section. Fig. 1b shows that the roughness of the matte surface was minimised (Rz = 1.7 μm) when the PEG concentration was 4 mg L^−1^, which was 26% lower than that of copper with no PEG. Adding 4 mg L^−1^ of PEG to the plating solution produced copper foils with optimum mechanical properties and a minimum surface roughness.

**Fig. 2:**
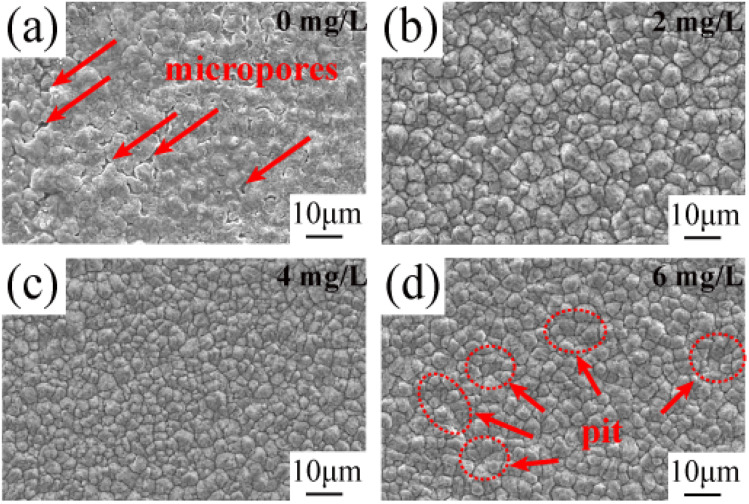
SEM images of the surfaces of copper foils prepared with different concentrations of PEG.

Fig. 2 shows the SEM morphology images of the matte surface of copper foils prepared with different concentrations of PEG. Without PEG, the copper foil surface presented many micropores (marked with red arrows in Fig. 2a). The micropores disappeared after the addition of 2 mg L^−1^ PEG, and the surface consisted of variously sized copper particles (Fig. 2b). At a PEG concentration of 4 mg L^−1^, the copper particles became fine and uniform (Fig. 2c). However, when the PEG concentration was 6 mg L^−1^, dents formed between the copper particles (the dotted circles in Fig. 2d), and the surface quality decreased. This may be because excess PEG became entangled with each other in the plating solution and adsorbed onto the copper foil during the electrodeposition process. This inhibited the reduction of Cu^2+^, resulting in pit formation. The 3D contour map of the matte surface showed that the contour undulation of the copper foil prepared with 4 mg L^−1^ of PEG was a minimum (ESI, Fig. S2†). The copper foil with 4 mg L^−1^ PEG in the plating solution had the best surface quality, which was consistent with the roughness variation shown in Fig. 1b.

### 3.2 Crystal orientation of copper foils

**Fig. 3:**
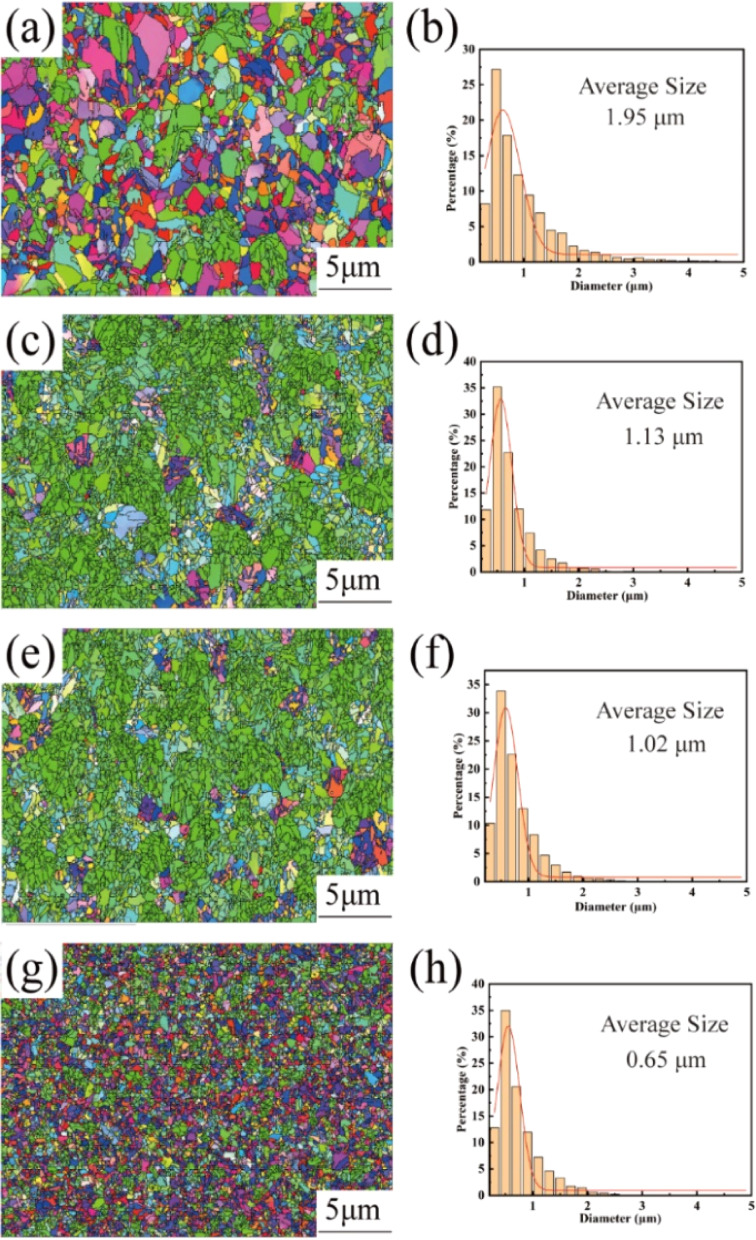
EBSD plots and corresponding grain size distributions on the surface of copper foils prepared with different PEG concentrations: (a and b) 0 mg L^−1^; (c and d) 2 mg L^−1^; (e and f) 4 mg L^−1^; (g and h) 6 mg L^−1^.

Fig. 3 shows the EBSD images and the corresponding grain size statistics of the copper foils prepared with different concentrations of PEG. In this figure, red indicates the (200) crystal plane, blue indicates the (111) crystal plane, and green indicates the (220) crystal plane. As shown in Fig. 3a, the copper foils without added PEG showed a weak specific crystal plane orientation. In contrast, at PEG concentrations of 2 and 4 mg L^−1^, the copper foils showed a wide range of green areas, indicating that they had a strong (220) crystal plane orientation (Fig. 3c and e). When the PEG was further concentration increased to 6 mg L^−1^, the strong (220) crystal plane orientation disappeared (Fig. 3g). This observation is consistent with the XRD test results in Fig. S3† of the ESI.† The average grain size of the copper foil without added PEG was relatively large, reaching 1.98 μm (Fig. 3b). With the increase in PEG concentration, the average grain sizes of the copper foils decreased to 1.13, 1.02, and 0.65 μm at 2, 4, and 6 mg L^−1^ PEG concentrations, respectively (Fig. 3d, f and h).

Based on the XRD data of the matte surface of the copper foil presented in ESI Fig. S3,† the texture coefficient *M*_*hkl*_ of the copper foil was calculated using eqn (1). The XRD pattern of the shiny surface of the copper foil is shown in ESI Fig. S4,† while the results are provided in Table 2. The texture coefficient *M*_220_ of the crystal plane of copper foil (220) increased gradually with the increase in PEG concentration. At 4 mg L^−1^ PEG, the *M*_220_ value of the crystal plane of copper foil (220) reached a maximum value of 86.1%. However, at 6 mg L^−1^ PEG, the *M*_220_ value of the crystal plane of (220) decreased to 57.1%.

**Table 2:** *M*_*hkl*_ values of copper foil crystal planes prepared with different concentrations of PEG

PEG concentration	*M*_111_	*M*_200_	*M*_220_	*M*_311_
0 mg L^−1^	23.5	16.9	39.3	20.3
2 mg L^−1^	6.6	2.5	82.6	8.3
4 mg L^−1^	4.9	2.7	86.1	6.3
6 mg L^−1^	13.7	6.3	57.1	22.9

### 3.3 Twin crystal structure of copper foils

**Fig. 4:**
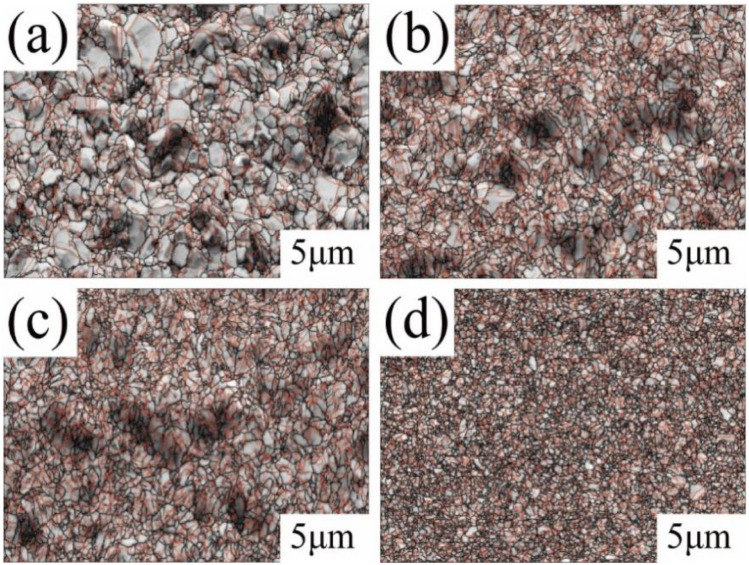
Distribution of ∑3 grain boundaries in copper foils with various PEG concentrations: (a) 0 mg L^−1^, (b) 2 mg L^−1^, (c) 4 mg L^−1^, and (d) 6 mg L^−1^.

Fig. 4 demonstrates the distribution of ∑3 grain boundaries in the copper foils based on EBSD data analysis. The ∑3 grain boundaries are a special type of grain boundary and are usually considered twin grain boundaries.^26–28^Fig. 4a shows that the number of twinned grain boundaries (marked in red) in the copper foils prepared without PEG was relatively small, accounting for only 45.9% of the total. At 2 and 4 mg L^−1^ PEG concentrations, the percentage of twinned grain boundaries increased to 50.6% and 51.9%, respectively (Fig. 4b and c). However, at a PEG concentration of 6 mg L^−1^, the number of twin boundaries decreased significantly to 35.9% (Fig. 4d).

**Fig. 5:**
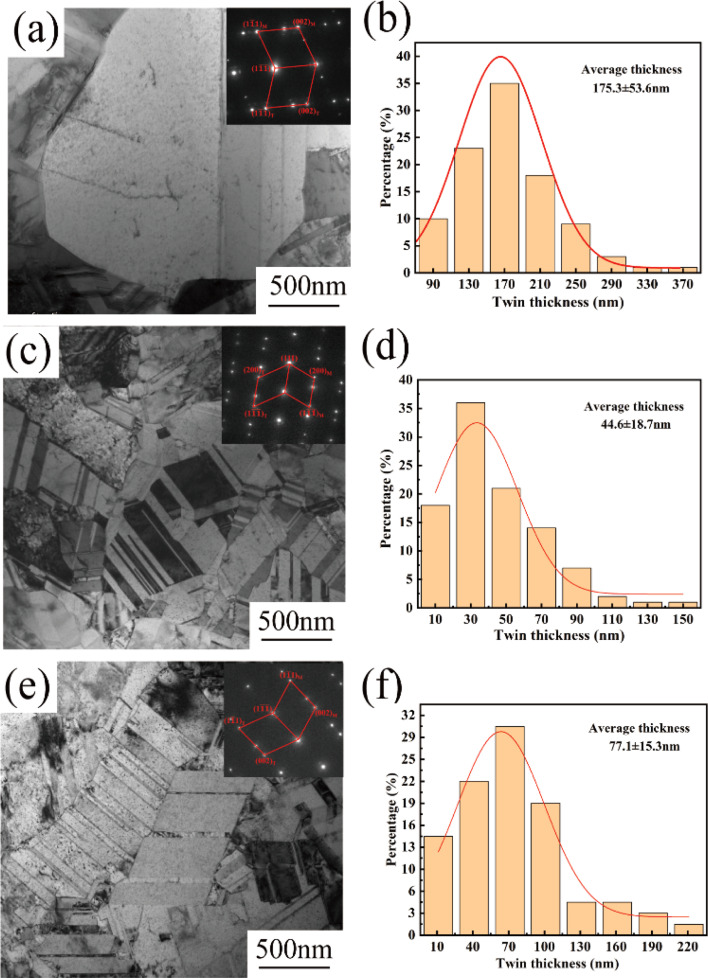
TEM images and the corresponding twin thickness distributions of copper foils prepared with various PEG concentrations: (a and b) 0 mg L^−1^, (c and d) 4 mg L^−1^ and (e and f) 6 mg L^−1^.

Fig. 5a, c and e show TEM images of the copper foil samples prepared with 0, 4, and 6 mg L^−1^ PEG concentration along the [001] Cu crystalline band axis. The corresponding SAED image is embedded within the TEM image. The diffraction spot index in the SAED pattern confirmed the presence of twinning in the copper foil samples. Fig. 5a shows that the copper foil prepared without PEG had coarse grains and fewer internal twins. The copper foils prepared with PEG concentrations of 4 and 6 mg L^−1^ contained a large number of twins inside (Fig. 5c and e). The sizes of nearly 100 twins were statistically analyzed based on the TEM images, and the distribution of the twin thickness was thus obtained (Fig. 5b, d and f). The results showed that the average twin thicknesses of the copper foils prepared with PEG concentrations of 0, 4, and 6 mg L^−1^ were 175.3, 44.6, and 77.4 nm, respectively. Direct current electroplating generates significant internal stress inside copper foil, which is usually released through the twinning mechanism induced by stress relaxation.^29^ When the PEG concentration reached 6 mg L^−1^, the residual amount of PEG inside the copper foil also increased. The residual PEG inside the copper foil hinders atom migration, decreasing the twin nucleation efficiency and increasing the thickness of the twin layer.^30^

**Fig. 6:**
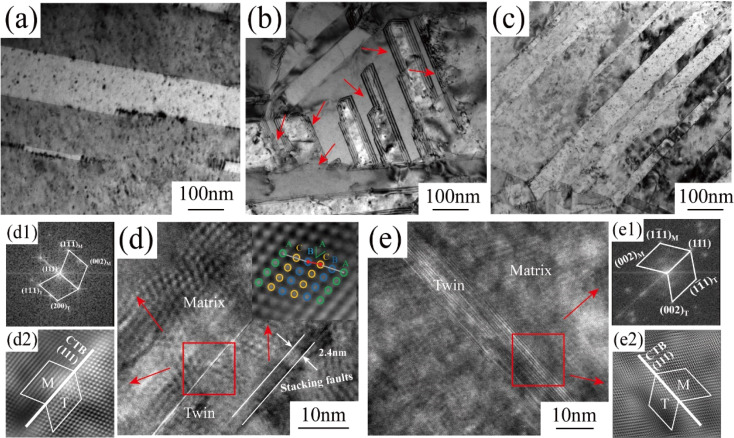
High-magnification TEM (a–c) and HRTEM (d and e) images of copper foils prepared by adding 0 mg L^−1^ (a), 4 mg L^−1^ (b, d, d1, and d2) and 6 mg L^−1^ (c, e, e1, and e2) PEG, and the corresponding plots obtained in the FFT mode (d1 and e1) and IFFT mode (d2 and e2).

To further investigate the differences in twinning structures of the copper foils prepared with different PEG concentrations, detailed observations of twinning were conducted using the HRTEM mode for the TEM images and fast Fourier transform (FFT) and inverse fast Fourier transform (IFFT) methods (Fig. 6). Fig. 6a shows that the copper foil prepared without PEG had coarse twin crystals and a thick twin layer. Fig. 6b shows the copper foils prepared with 4 mg L^−1^ PEG concentration with a clear layer structure at the boundary of the twins. In the HRTEM image (Fig. 6d), the structure at the twinning boundary is a stacking fault with a length of approximately 2.4 nm. The IFF image of the stacking fault region is embedded in Fig. 6d. It is clearly visible from the figure that the stacking of copper atoms was incorrect, with the A-layer atoms not stacked properly within the lattice. Consequently, the type of stacking fault is intrinsic, involving the displacement of atoms by one Burgers vector. In contrast, no such stacking fault was observed at the twinning boundary of the copper foils prepared with 6 mg L^−1^ PEG (Fig. 6c and e). From the FFT and IFFT images in Fig. 6e1 and e2, it could be found that the twin boundaries of both copper foil samples showed the characteristics of ∑3{111} coherent twin boundaries, which is in accordance with the results of previous studies.^27,31^

### 3.4 Electrochemical behavior of the plating solution

**Fig. 7:**
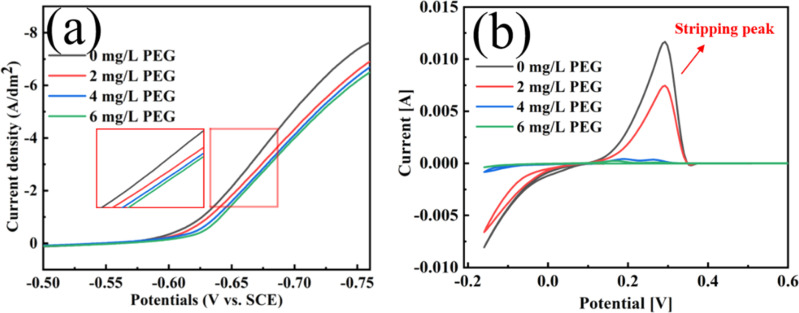
LSV (a) and CV (b) curves from different concentrations of PEG in the plating solution.

Fig. 7 shows the linear sweep voltammetry (LSV) and cyclic voltammetry solubility (CVs) test curves for plating solutions containing various PEG concentrations. The deposition potential grew increasingly negative as the PEG concentration increased (Fig. 6a) This shift to a negative potential indicates enhanced cathodic polarisation, which inhibits the electrodeposition of Cu^2+^. An analysis of the CVs curves in Fig. 7b showed that the stripping peak area decreased by 37.2%, 95.6% and 98.1% when the PEG concentrations were 2, 4, and 6 mg L^−1^, respectively. This decrease in the stripping peak area indicates that the amount of copper deposited on the electrode was reduced and the copper electrodeposition was inhibited. This is consistent with the results of the chronopotentiometry (CP) curves in Fig. S5 in the ESI.†

### 3.5 Analysis of the mechanical properties of copper foil

Adding PEG increased the cathodic polarisation and raised the deposition overpotential. Therefore, the nucleation growth model is still applicable.^32^ The relationship between nucleation rate and cathodic polarisation overpotential can be expressed by the following equation:^33^2
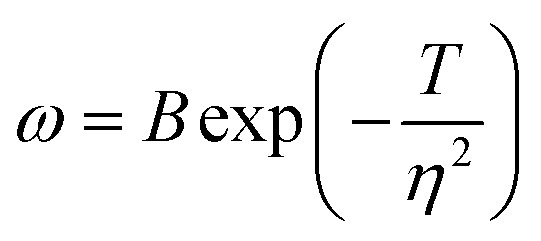
where *ω* is the nucleation rate, *B* is a constant related to the material, *T* is the temperature, and *η* is the cathodic overpotential. As eqn (2) shows, the rate of formation of new nuclei on the electrode surface increased exponentially with the increase in cathodic overpotential. The production of many nuclei forms fine grains in the copper foil, which increases its tensile strength and elongation due to fine-grain strengthening. Notably, when the PEG concentration was increased to 6 mg L^−1^, the tensile strength of the copper foil decreased, even though the grains became finer (Fig. 1a). This suggests that the tensile strength of copper foils is not controlled by a single fine-grain strengthening, and that other factors affect their tensile strength.

**Fig. 8:**
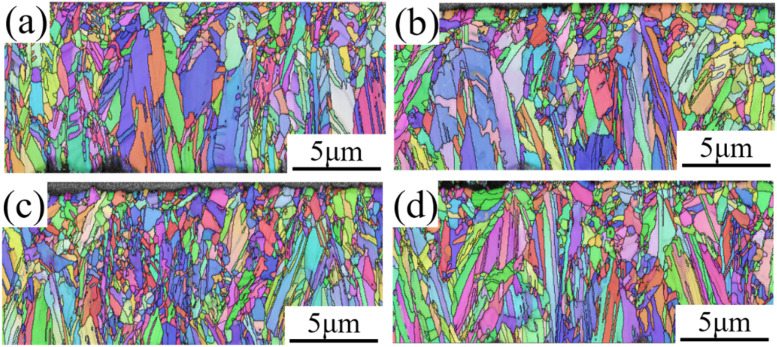
Cross-sectional EBSD images of copper foils prepared with different PEG concentrations: (a) 0 mg L; (b) 2 mg L; (c) 4 mg L; (d) 6 mg L^−1^.

Fig. 8 shows EBSD images of cross-sectional views of copper foil prepared with different PEG concentrations. The figure reveals that the copper foil cross-section was primarily composed of columnar crystal grains aligned along the growth direction. With increasing PEG concentration, the columnar crystal grains became finer. The columnar grain sizes of copper foils prepared with 0, 2, 4, and 6 mg L^−1^ PEG were 0.527, 0.482, 0.449, and 0.441 μm, respectively. This demonstrates that the increase in cathodic polarization due to PEG concentration can also refine the columnar grains on the copper foil cross-section, and enhance the mechanical properties of the copper foil *via* fine-grain strengthening. Fig. 8c shows the minimal variation in grain size, resulting in the highest number of grains along the same thickness direction, which may also contribute to the enhancement of the strength of the copper foil.^34^

Research has shown that the ratio (*t*/*d*) between the thickness (*t*) of the metallic foil materials and the average grain size (*d*) in the thickness direction significantly impacts the mechanical properties of metal foil.^35–37^ When the *t*/*d* ratio approaches 1, the mutual influence between the foil thickness and grain size becomes a primary determinant of the material's mechanical properties.^38^ However, in this study, it was observed that the cross-section of copper foil was primarily composed of elongated columnar grains (Fig. 8), making it inappropriate to directly use the length of the columnar grains to calculate the *d* value. Additionally, the high density of twin grains within the columnar grains also affects the calculation of the *d* value.^39^ Therefore, this study concludes that the *t*/*d* ratio is not suitable as an indicator for assessing changes in the mechanical properties of copper foil.

During the electrodeposition of copper, the growth of the crystal planes needs to overcome their respective energy barriers.^40^ According to the Bravais law, the growth rate of each crystal plane during the deposition process follows the order of (200) > (220) > (111). Zhang *et al.*^26^ showed that ether functional groups exhibited obvious selectivity for the three main crystal plane of copper, and the adsorption energy of the (111) crystal plane was significantly higher than that of the other two crystal plane. This property effectively inhibited the growth of the (111) crystal plane. The addition of PEG, a polyether organic compound with ether functional groups, can effectively promote the selective orientation of the (220) crystal plane in copper foils. According to Zhang *et al.*,^41^ in face-centered cubic crystals, the surface energy of the (220) crystal plane is higher than that of the (100) and (111) crystal planes. The surface energy is the energy per unit area required to divide a crystal into two semi-infinite bodies. Therefore, the (220) crystal plane requires the highest energy for deformation. Fan *et al.*^42^ calculated the Schmid factor values for face-centered cubic cells and showed that on the (220) crystal plane, the least amount of slip could be activated under different tensile orientations, indicating that it had the maximum deformation resistance. Therefore, the higher the *M*_220_ of the (220) crystal plane in the copper foil, the higher its tensile strength. Table 2 shows that the *M*_220_ value of the copper foils was largest at 86.1% when the PEG concentration was 4 mg L^−1^. At this point, the copper foil possessed the highest tensile strength. When the PEG concentration was increased to 6 mg L^−1^, the *M*_220_ of the prepared copper foil decreased significantly to 57.1%. This phenomenon occurs because excess PEG molecules are electrostatically adsorbed onto the (220) crystal plane, leading to inhibition of the (220) plane growth, consequently reducing the orientation intensity of the (220) plane. The tensile strength of the copper foils also decreased (Fig. 1a).

The relationship between the twin thickness and mechanical strength of copper foil is similar to the relationship between the grain size and strength in the Hall–Petch relationship.^43^ According to the Hall–Petch formula, reducing the twin thickness of copper foil can effectively improve its mechanical properties.^44,45^ As Fig. 5 shows, the twin thickness of copper foils prepared with a concentration of 4 mg L^−1^ of PEG was smaller than that of samples prepared with 6 mg L^−1^. A smaller twin thickness means that there are more twin boundaries within the grains, and the twin boundaries can effectively impede the movement of dislocations, thus enhancing the tensile strength of the copper foil. The high density of twin boundaries inside the copper foil allows the plastic deformation to be dispersed in a smaller area, and the deformation is more uniform, which effectively increases elongation. In addition, the stacking faults in the copper foils with 4 mg L^−1^ of PEG strengthened the twin boundaries so that the obstruction of the boundaries to the dislocations increased. This further improved the tensile strength of the copper foils. Therefore, the 4 mg L^−1^ PEG concentration was most favorable for reducing the twin size and for forming stacking faults at the twin boundaries to obtain the best tensile strength and elongation (Fig. 1b).

## 4. Conclusion

This study aimed to investigate the relationship between the PEG concentration and the microstructure and mechanical properties of copper foil. The results showed that the optimum PEG concentration was 4 mg L^−1^, which resulted in the highest tensile strength (433.2 MPa) and elongation (2.2%) in the copper foil, in addition to the best surface quality and the lowest surface roughness (Rz = 1.7 μm). This concentration of PEG also resulted in a maximum *M*_220_ of 86.1% on the crystal plane of the copper foil (220), and the lowest average twin thickness (44.6 nm), while the formation of stacking faults strengthened the twin boundaries. As the PEG concentration increased, the cathodic polarisation effect was enhanced, and the nucleation rate increased, thus refining the copper foil grain size. When the PEG concentration was 6 mg L^−1^, the average grain size decreased to 0.65 μm, and the fine-grain strengthening effect increased. However, the *M*_220_ of the (220) crystal plane orientation of the copper foil decreased to 57.1%, and the twin thickness increased to 77.1 nm, resulting in a significant decrease in tensile strength. This indicates that the high orientation of the (220) crystal plane and the high density of twin boundaries strengthened by stacking faults have a greater effect on the strengthening of the copper foils than the strengthening of the fine crystals of the copper foils. These results indicate that PEG concentration plays a key role in the surface quality, microstructure, and mechanical properties of copper foils.

## Data availability

The authors declare that all the experimental data are true and original and can be repeated from the work reported in this paper.

## Conflicts of interest

The authors declare that they have no known competing financial interests or personal relationships that could have appeared to influence the work reported in this paper.

## Supplementary Material

RA-014-D4RA06688J-s001
