# Supplementary material for: Electrodeposition, microstructure and characterization of high-strength, low-roughness copper foils with polyethylene glycol additives
Source: RSC Adv. 2024 Dec 3;14(51):38268–78. doi: 10.1039/d4ra06688j (PMC11612898; doi:10.1039/d4ra06688j)
Supplement: RA-014-D4RA06688J-s001 [file RA-014-D4RA06688J-s001.pdf]

Supplementary Information (SI) for RSC Advances.
This journal is © The Royal Society of Chemistry 2024

Electrodeposition, microstructure and characterization of high-strength, low-roughness copper foils with polyethylene glycol additives

Jian Huang , Ning Song, Mingwei Chen, Yunzhi Tang*, Xiaowei Fan

Jiangxi Province Key Laboratory of Functional Crystalline Materials Chemistry, School of Chemistry and Chemical Engineering, Jiangxi University of Science and Technology, Ganzhou 341000, China

* Corresponding Authors:

*E-mail: tangyunzhi75@163.com (Yunzhi Tang)

Figure S1 shows cross-sectional EBSD images of copper foils prepared with 4 mg/L PEG at 1 hour and 3 hours after electroplating completion. As can be seen in Figure S1, the copper foil's crystal structure remains highly stable at both 1 hour and 3 hours post-electroplating. No self-annealing behavior is observed in the copper foil within the first 3 hours after electroplating.

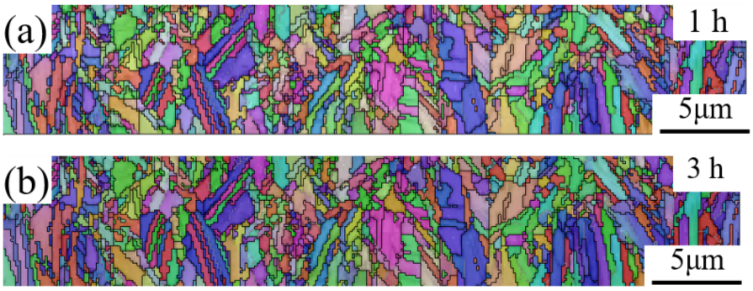

Figure S1. EBSD images of copper foil prepared with 4mg/LPEG after 1 hour (a) and 3 hours (b) of electroplating completion

Figure S2 shows the 3D profiles of copper foils with different PEG concentrations. Rp denotes the distance between the highest peak line and the lowest

valley line of the largest profile, which can be used to reflect the surface roughness of the copper foils in the test area. As can be seen in Figure S2, the surface of the copper foil has concave (blue) and convex (red) 3D morphology. According to the SEM photo in Figure 2(a), it can be seen that the concave region is the microporous region of the copper foil and the convex region is the region where the copper particles are located. At this time, the cross-section profile of the copper foil fluctuates with a high Rp of 8.7 μm and high surface roughness (Figure S2(b)). With the increase of PEG concentration to 2 mg/L, the cracks on the surface of the copper foil were reduced, and the surface of the copper foil was mainly composed of copper particles with three-dimensional morphology (Figure S2(c)). The surface roughness of the copper foil was improved, and the Rp value was reduced to 3.1 μm (Figure S2(d)). With a PEG concentration of 4 mg/L, the surface of the copper foil consisted of uniformly dense copper particles with three-dimensional morphology (Figure S2(e)). The cross-sectional profile of the copper foil has less undulation, and the Rp value decreases to a minimum of 2.5 μm to obtain the best surface quality (Figure S2(f)). When the PEG concentration was further increased to 6 mg/L, the gap between the copper particles on the surface of the copper foil became larger (Figure S2(g)). This led to larger fluctuations in the surface profile of the copper foil, an increase in the Rp value to 2.8 μm, and a decrease in the surface quality of the copper foil (Figure S2(h)).

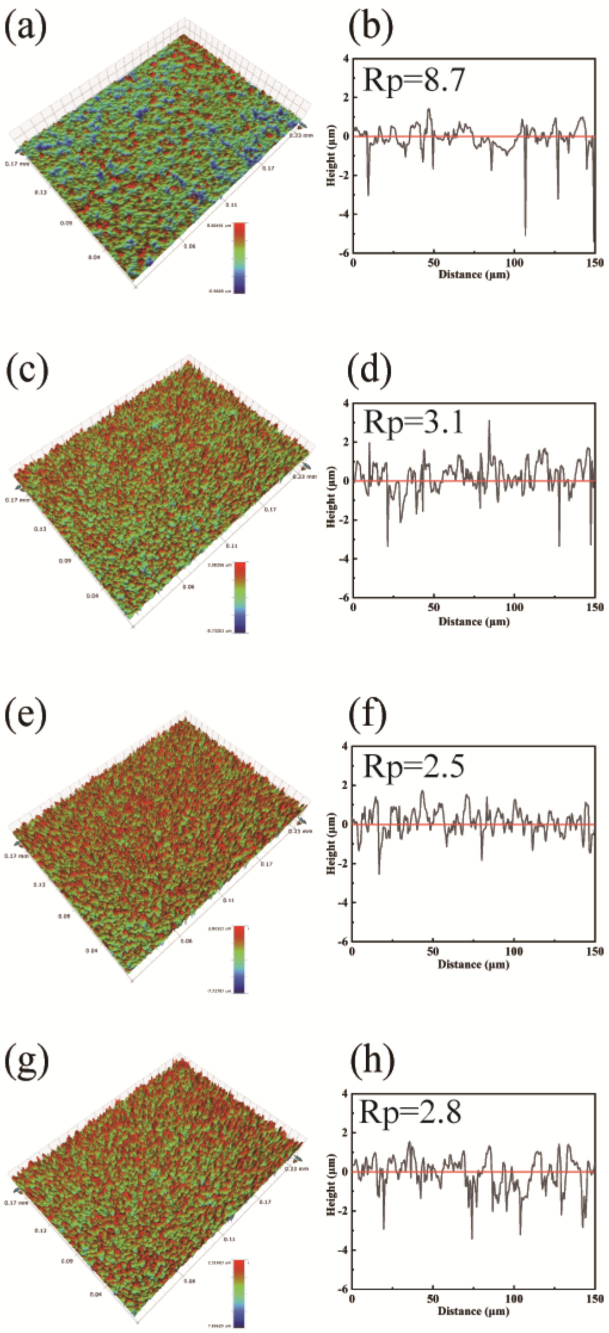

Figure S2 3D morphology and corresponding cross-sectional profile changes of copper foils with different PEG concentrations: (a, b) 0 mg/L; (c, d) 2 mg/L; (e, f) 4 mg/L; (g h) 6 mg/L.

Figure S3 shows the XRD patterns of copper foils prepared with different concentrations of PEG. From the figure, it can be seen that the strongest diffraction

peak of the copper foil prepared without PEG is from the (111) crystal plane. When 2 mg/L PEG was added, the maximum diffraction peak of the copper foil changed from the (111) crystal plane to the (220) crystal plane. The copper foil showed a strong optimum orientation of the (220) crystal plane. When the PEG concentration was further increased to 4 mg/L, the copper foil still showed a strong (220) crystal plane selective orientation. However, when the PEG concentration reached 6 mg/L, the intensity of the diffraction peaks of the (220) crystal plane of the copper foils decreased significantly.

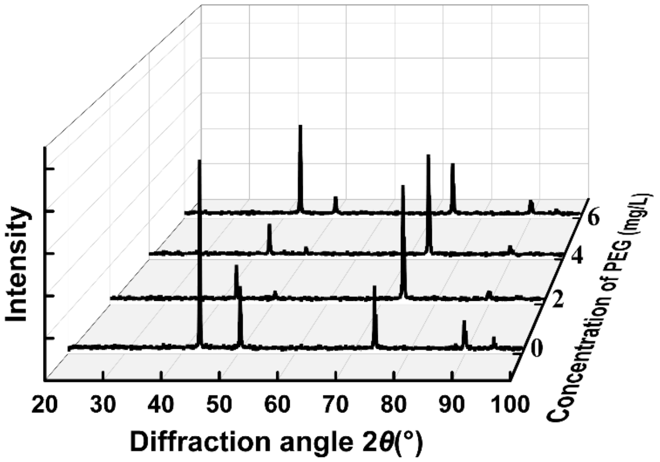

Figure S3 XRD patterns of copper foil prepared with different concentrations of PEG

Figure S4 shows the XRD patterns of the bright surfaces of copper foils prepared with different concentrations of PEG. As can be seen from the figure, the peak shapes of the XRD patterns of the samples used are basically consistent. According to the theory of electrodeposition epitaxial growth, in the initial stage of electro-crystallization, copper atoms grow along the crystal lattice of the titanium substrate. This process is largely dependent on the crystal structure of the titanium substrate and

is minimally influenced by the additives. In this study, samples were prepared using the same titanium plate, so the XRD patterns of the shiny side of the copper foils in contact with the titanium plate are essentially the same under different PEG concentrations.

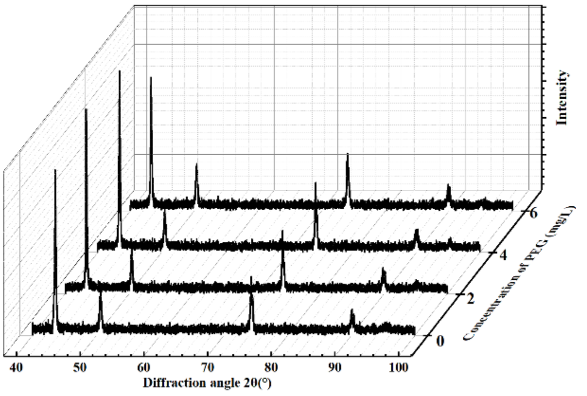

Figure S4. XRD patterns of copper foil shiny side prepared by different concentrations of PEG

The potential variation of electrodeposited Cu at constant current density was analyzed using the chronopotential method. As shown in Figure S5, all curves exhibit increasing potential after initiation, reaching a steady state after approximately 40 seconds. This is due to the control step of electrodeposited Cu transitioning from nucleation to diffusion of Cu^{2+}. From the curves, it can be seen that the addition of PEG leads to a decrease in the steady state potential. This is attributable to the polarizing effect of PEG, necessitating a higher potential to maintain a given steady state current. Thus, the steady state potential difference can reflect the magnitude of the polarization potential. The steady state potential differences caused by PEG concentrations of 2 mg/L, 4 mg/L, and 6 mg/L were 0.013 V, 0.071 V, and 0.078 V,

respectively. The steady state potential difference caused by 4 mg/L PEG was notably larger than that of 2 mg/L PEG, and was not substantially different from that of 6 mg/L PEG.

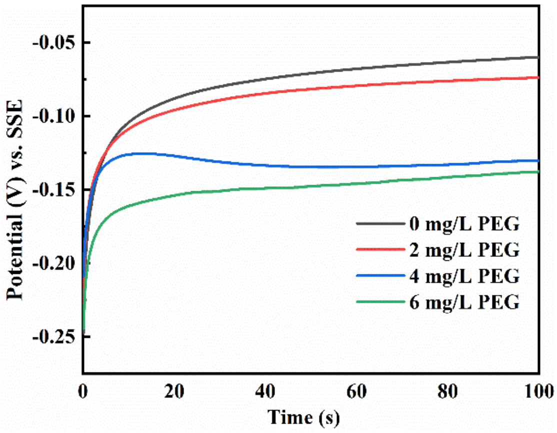

Figure S5 CP curves of different concentrations of PEG in plating solution
